# Human umbilical cord mesenchymal stem cells derived exosome shuttling mir-129-5p attenuates inflammatory bowel disease by inhibiting ferroptosis

**DOI:** 10.1186/s12951-023-01951-x

**Published:** 2023-06-12

**Authors:** Zhiping Wei, Sanhua Hang, Dickson Kofi Wiredu Ocansey, Zhaoyang Zhang, Bo Wang, Xu Zhang, Fei Mao

**Affiliations:** 1 Key Laboratory of Medical Science and Laboratory Medicine of Jiangsu Province, School of Medicine, Jiangsu University, 301 Xuefu Road, Zhenjiang, Jiangsu 212013 P.R. China; 2 The People’s Hospital of Danyang, Affiliated Danyang Hospital of Nantong University, Zhenjiang, Jiangsu 212300 P.R. China; 3 Clinical Lab, Taicang Hospital of Traditional Chinese Medicine, Suzhou, Jiangsu 215400 P.R. China

**Keywords:** Mesenchymal stem cell-derived exosomes, Ferroptosis, miR-129-5p, Inflammatory bowel disease, ACSL4, Lipid peroxidation

## Abstract

**Background:**

Ferroptosis, a unique form of non-apoptotic cell death, is dependent on iron and lipoperoxidation, and has been shown to be associated with the pathogenesis of inflammatory bowel disease (IBD). Human umbilical cord mesenchymal stem cell-derived exosomes (hucMSC-Ex) are involved in cell survival, immune conditioning, and damage repair. However, the relationship between hucMSC-Ex, IBD, and ferroptosis is unknown. This paper explores the role of hucMSC-Ex in the repair of IBD through the regulation of the ferroptosis signaling pathway.

**Results:**

In this study, we used small RNA sequencing to find that miR-129-5p was highly expressed in hucMSC-Ex, and by predicting its targeting to ACSL4, we verified the effect of miR-129-5p on mice IBD in vitro and human colonic epithelial cells (HCoEpiC) in vivo. We found that miR-129-5p reduces ferroptosis in intestinal epithelial cells by targeting ACSL4 to repair IBD, which provides new strategies for the prevention and treatment of IBD.

**Conclusion:**

In conclusion, our results demonstrate that hucMSC-Ex relieves IBD by targeting ACSL4 with miR-129-5p to inhibit lipid peroxidation (LPO) and ferroptosis, reducing intestinal inflammation and repairing damages.

**Graphic abstract:**

Mechanism of hucMSC-Ex inhibiting ferroptosis in intestinal epithelial cells. System Xc^−^ mediates the transport of extracellular cystine into the cell, which gets reduced to cysteine to participate in GSH-mediated metabolism. GPX4 strongly inhibits ferroptosis by helping scavenge reactive oxygen species. The depletion of GSH correlates with decreased GPX4, and the imbalance of the antioxidant system leads to the formation of toxic phospholipid hydroperoxide, which promotes the occurrence of ferroptosis with the participation of irons. HucMSC-Ex has the ability to relieve GSH and GPX4 depletion and repair the intracellular antioxidant system. Ferric ions enter the cytosol through DMT1 and participate in lipid peroxidation. HucMSC-Ex can reduce the expression of DMT1 and alleviate this process. HucMSC-Ex-derived miR-129-5p targets ACSL4 and reduces the expression of ACSL4, an enzyme that mediates the conversion of PUFAs into phospholipids in intestinal epithelial cells, and is a positive regulator of lipid peroxidation. Abbreviations: GSH, glutathione; GPX4, glutathione peroxidase 4; GSSG, oxidized glutathione; DMT1, divalent metal transporter 1; ACSL4, acyl-CoA synthetase long-chain family member 4; PUFAs, polyunsaturated fatty acids; ALOXs, lipoxygenases; CoA, coenzyme A; PL, phospholipid; PLOOH, hydroperoxides, LOH, phospholipid alcohols; LPO, lipid peroxidation.

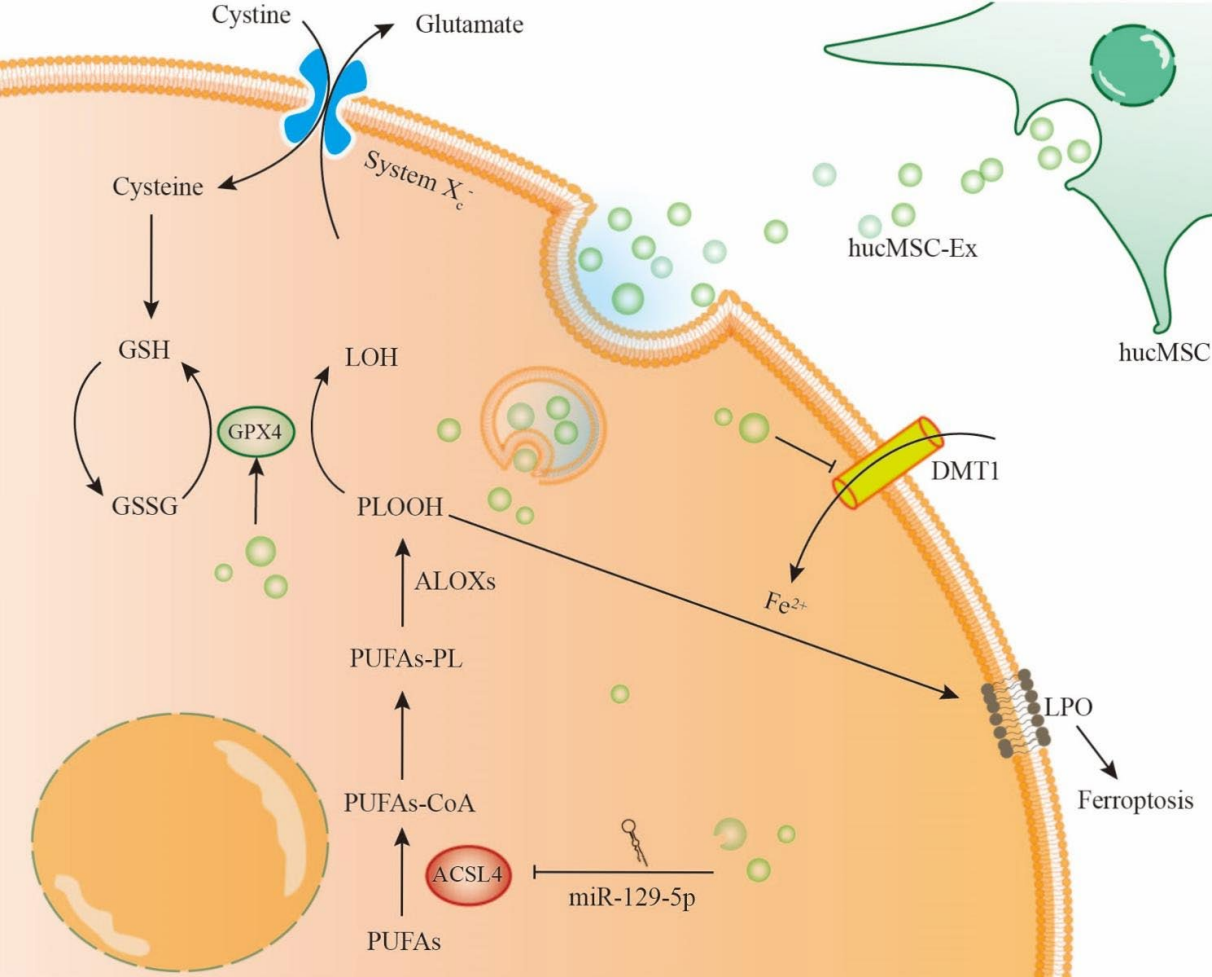

## Introduction

Inflammatory bowel disease (IBD) is a chronic and persistent intestinal inflammatory disease with unknown etiology. It is usually believed to be related to intestinal microbiota imbalance, environmental, and genetic factors. IBD is mainly classified as ulcerative colitis (UC) and Crohn’s disease (CD) and has evolved with industrialization into a global disease, seriously affecting the living standards and well-being of patients [1]. Currently, effective clinical prevention and treatment of IBD remain a challenge, with existing treatments offering limited remission outcomes. Thus, the results of epidemiological and clinical studies indicate the necessity to develop new treatment methods to improve the therapeutic outcome in IBD patients [2].

Mesenchymal stem cells (MSCs), as ideal seed cells, have achieved significant efficacy in the treatment of IBD. Studies have shown that MSCs are mainly involved in tissue damage repair through the paracrine pathway, and extracellular vesicles (EVs) are the main effector components of MSCs to exert their paracrine effects. EVs are composed of exosomes and microvesicles (MVs), which are involved in physiological and pathological processes such as cell survival, immune regulation, angiogenesis, and tissue regeneration [3, 4]. Exosomes are small extracellular bilayer vesicles with diameters of 30–150 nm and contain bioactive substances similar to the components of their source cells, which makes them have similar physiological functions as the source cells [5]. Mesenchymal stem cell-derived exosomes (MSC-Ex) have been extensively investigated for their potential in regenerative medicine, where a large number of studies have shown that MSC-Ex exerts great potential in the treatment of various diseases and tumors [6, 7]. Exosomes derived from MSCs from different sources usually have similar functions and the potential for anti-inflammation and reparation. Exosomes derived from umbilical cord mesenchymal stem cells (hucMSC-Ex) have attracted wide attention due to their non-invasive acquisition, and exert a powerful repair potential in the treatment of IBD [8, 9]. HucMSC-Ex has been reported to modulate Th2 and TH17 cell responses in mesenteric lymph nodes, down-regulate proinflammatory factors, and restore mucosal barrier function [10]. Other studies have shown that hucMSC-Ex could alleviate experimental colitis in mice through PEG2-mediated macrophage polarization [11]. The bioactive substances contained in exosomes, like nucleic acids and proteins, are key to their function. Although hucMSC-Ex has been shown to have tissue regeneration and repair effects in IBD, the specific mechanisms still need to be explored.

MicroRNAs (miRNAs) are short-chain RNA molecules that regulate protein transcription in the translation stage and exert their biological effects by targeting different mRNAs. They induce gene silencing by complementarily targeting the 3 ‘UTR of the mRNA [12]. This property has led to an interest in the prospects of miRNA therapy. Through the identification and modification of miRNAs, targeted vector systems have been designed to enhance the therapeutic efficacy of miRNAs [13]. Exosomes, as natural delivery systems, have been found to contain a variety of bioactive miRNAs that can be used in the treatment of diseases. For example, MSC-Ex-derived MiR-146 and miR-155 act as major immune response regulators in rheumatoid arthritis (RA) [14]. Moreover, MSC-Ex reduces myocardial ischemia-reperfusion injury in mice by shuttling miR-182 to aid the polarization of macrophages, facilitating the generation of the anti-inflammatory M2 phenotype [15]. Thus, whether hucMSC-Ex acts through the large number of miRNAs it contains requires further verification.

In addition to the two important forms of cell death, apoptosis and necrosis, non-apoptotic death, including necrosis, pyroptosis, ferroptosis, and other gene-regulated death processes, has attracted research attention [16]. Ferroptosis is a unique form of nonapoptotic cell death that is iron-dependent and characterized by increased lipid peroxidation (LPO), which is initiated by nonenzymatic and enzymatic mechanisms [17, 18]. In this process, ferric iron (Fe^3+^) enters the endosome of the cells and is reduced to divalent iron (Fe^2+^). Divalent metal transporter 1 (DMT1) mediates the release of iron ions from endosomes to the cytoplasm. Excessive iron accumulation produces reactive oxygen species (ROS) through the Fenton reaction and destroys cells. LPO is under steady-state metabolic control and peroxidation occurs only when the indispensable constraint conditions are met and the anti-peroxidation mechanism breaks down. The activity of selenoperoxidase glutathione peroxidase 4 (GPX4) is the cornerstone of the anti-peroxidation defense, which utilizes glutathione (GSH) synthesis to combat LPO [19]. Depletion of GPX4 represents the collapse of the antioxidant system, where GSH depletion and GPX4 suppression can cause dysregulation of the antioxidant system of cells, resulting in the accumulation of toxic LPO [20]. LPO products are produced by the reaction between ROS and polyunsaturated fatty acids (PUFAs) in cell membranes. Genes involved in this process include lysophosphatidylcholine acyltransferase 3 (LPCAT3) and acyl-CoA synthetase long-chain family member 4 (ACSL4) [17, 21–23]. ACSL4 is an important isoenzyme in PUFA metabolism that determines ferric iron death sensitivity. Studies have shown that ACSL4 knockout has a protective effect on stroke [24]. There are also studies that show that pre-inhibition of ACSL4 alleviates ferroptosis and cell death in intestinal ischemia-reperfusion injury [25].

IBD is thought to be associated with diets rich in PUFAs because of the increased incidence of IBD in developed Western countries and the increasing incidence in developing countries, where people consume a PUFA-rich Western diet [26–28]. Studies have shown that the intestinal epithelium of IBD patients is characterized by inflammation, resulting in intestinal barrier damage, and ferroptosis plays an important role in the process of intestinal epithelial death [27]. Researchers have found that the intestinal epithelial cells of IBD patients have the basic characteristics of ferroptosis, such as the deposition of iron particles, glutathione depletion, GPX4 inactivation, lipid peroxidation, and ROS generation [28–30]. In addition, the inhibition of ferroptosis has significance for the remission of IBD [31, 32], as reported in experimental colitis in mice [33]. These results suggest that ACSL4-related LPO is linked to the damage of diseased tissues, which may be caused by ferroptosis. Based on the observation that the inhibition of ferroptosis-related pathways can alleviate intestinal inflammation, it is reasonable to infer that inhibiting the ACSL4-related pathway can mitigate intestinal injury in IBD. Current studies have shown that hucMSC-Ex can alleviate IBD, but the specific mechanism behind this effect has not been fully elucidated. Therefore, we sought to investigate whether hucMSC-Ex could inhibit intestinal epithelial ferroptosis by targeting ferroptosis mediators and pathways. In this study, we verify the reparative effect of hucMSC-Ex on IBD in mice and reveal the effect of LPO on IBD injury and the mechanism by which hucMSC-Ex inhibits ACSL4 to prevent LPO in the repair of IBD. These results provide a better understanding of the repair mechanism of hucMSC-Ex and a basis for the clinical treatment of IBD.

## Material and method

### Isolation and culture of hucMSC

Human umbilical cords were cut into 1mm^3^ pieces and cultured in α-MEM (hyclone) medium supplemented with 15% fetal bovine serum (FBS; Excell Bio) and 1% Penicillin-Streptomycin-Amphotericin B Solution (Procell), with the solution changed every other day. Around 7–10 days, hucMSCs were seen migrating from the tissue. HucMSC was cultured in α-MEM supplemented with 10% FBS and 1% Penicillin-Streptomycin-Amphotericin B Solution. After 24 h of culture, the supernatant was discarded and the medium was replaced with fresh 10% α-MEM. The FBS supplemented with the medium was processed by hyper-centrifugation to remove the exosomes. The supernatant was collected after 48 h of culture for subsequent experiments.

### Extraction, identification, and transfection of hucMSC-Ex

After a series of centrifugation to remove dead cells and debris, the collected cell supernatant was placed in a 100 kDa ultrafiltration tube (Millipore) for further centrifugations. The supernatant obtained by centrifugation was further centrifuged at 100,000 g, then the supernatant was discarded, resuspended in PBS, filtered through a 0.22 μm filter (Millipore), and stored at -80 ° C for later use. NanoSight nanoparticle tracking analysis (NTA) was used to determine the particle number and particle size of exosomes. The morphology of exosomes was observed by transmission electron microscopy (TEM). Western blot was used for the identification of exosome surface markers CD9, CD81, Calnexin, and ALIX (1:500; Affinity). Transfection of exosomes was performed using Exo-Fect™ Exosome Transfection Reagent (SBI) according to the manufacturer’s instructions.

### Cell culture and transfection

Human colonic epithelial cells (HCoEpiC) were purchased from Nanjing Saihongrui Biotechnology Co., Ltd. HCoEpiC and human embryonic kidney cell line (HEK293T) were cultured in DMEM medium (hyclone) with 10% FBS and 1% Penicillin-Streptomycin-Amphotericin B Solution, and incubated at 37℃ with 5% CO^2^. The transfection system was configured with mimics (Gamma gene) and Lipofectamine 2000 (Invitrogen). 1 × 10^6^ HCoEpiC were plated in a 6-well plate. When the cell density reached 60-80%, the supernatant was changed into a serum-free DMEM medium, and the above transfection system was added. After 4 h of incubation in the dark, the medium was changed into a complete medium for another 24 h. Cells were harvested for subsequent experiments.

### Cell viability assays

HCoEpiC were seeded into a 96-well plate at 1,500 cells/well with 100 µl of 10% FBS DMEM. Cells were divided into the NC group, LPS group, and hucMSC-Ex group. The NC group was replaced with fresh 10% FBS DMEM, while the LPS and hucMSC-Ex groups were replaced with 10% FBS DMEM containing 2 µg/ml LPS (Sigma). The hucMSC-Ex group was treated with an additional 400 µg/ml exosomes and all setups were incubated for 24 h. Cell viability was determined using a CCK-8 reagent (Vazyme).

### Establishment of IBD mouse models

Male BABL/C mice (6–8 weeks old, 17-23 g) were purchased from the Animal Research Center of Jiangsu University (Jiangsu, China). Relevant studies were carried out with the approval of the Ethics Committee and the Laboratory Animal Management and Use Committee of Jiangsu University. The mouse model of colitis was established by freely drinking 3% dextran sodium sulfate (DSS). Mice were randomly divided into three groups: NC group, DSS group, and hucMSC-Ex group. Mice in the NC group were given tap water, while mice in the DSS group and the hucMSC-Ex group were given tap water supplemented with 3% DSS. On the third, sixth, and ninth days after drinking 3% DSS, the NC and DSS groups were injected with sterile PBS, and the hucMSC-Ex group was injected with 1 mg/20 g weight of hucMSC-Ex. The mice were weighed at the same time every morning, and their living conditions and stool traits were observed. On day 10, mice were sacrificed and colorectal tissues were extracted for further experiments.

### Determination of T-GSH

Colonic tissues of each group were harvested and rinsed twice with pre-cooled PBS buffer, and the tissues were accurately weighed for later use. Colon tissue homogenate was prepared according to the instructions of the T-GSH detection kit (Jincheng, Nanjing). The concentration of T-GSH was obtained by measuring the absorbance at 405 nm with a microplate reader.

### Western blot

Total proteins of cells and tissues were extracted with RIPA lysate (Solarbio) supplemented with protease inhibitors PMSF (Solarbio). The concentration of total proteins was measured using the BCA(Vazyme). The total protein was mixed with loading buffer (Life Technologies) at a ratio of 3:1 and boiled at 100℃ for 8 min. According to the concentration, the protein samples were added to the 10% SDS polyacrylamide gel (Yamei, Shanghai) and the proteins were separated by electrophoresis according to their different molecular weights. The protein on the gel was transferred to polyvinylidene fluoride PVDF membrane (Millipore) and blocked in 5% skim milk for one and a half hours, followed by incubation with primary antibodies at 4℃ for one night. After washing with 1× TBS/T buffer, the membranes were incubated with the secondary antibody for 1 h at 25℃. The protein bands were visualized with a chemical gel imaging system (GE). The primary antibodies used were: anti-CD9 (1:500; Affinity), anti-CD81 (1:500; Affinity), anti-Alix (1:500; Affinity), anti-Calnexin (1:500; Affinity), anti-ACSL4 (1:1000; Abcam), anti-GPX4 (1:500; Affinity), anti-DMT1 (1:500; Affinity), anti-COX2 (1:500; CST) anti-PCNA (1:1000; Santa), anti-β-actin (1:1000; Santa), anti-Claudin-1 (1:1000; Proteintech), and anti-Occludin (1:5000; Proteintech). The secondary antibody used were: Goat anti-rabbit IgG, FITC conjugated (1:200; proteintech), and Goat anti-rabbit IgG (1:10000; Immunoway).

### qRT-PCR analysis

Total mRNA from tissues and cells was extracted by Trizol (Vazyme). The mRNA was reverse transcribed using the reverse transcription kit (Vazyme). The real-time fluorescence quantification PCR (qRT-PCR) was performed to measure the transcript abundance of the genes with Synergy Brands (SYBR) Green detection(Vazyme). The relative expression level of the target gene was calculated using mRNA expression of β-actin as a control. The sequences of primers used are listed in Table 1.

**Table 1:** Primer sequence

Gene	Primer Sequence
human-DMT1/SLC11A2-F	CATCCTCACATTTACGAGCTTG
human-DMT1/SLC11A2-R	CCAACCCAAGTAGAACACAAAG
mouse-DMT1/Slc11a2-F	TTTTGGACAAATATGGCTTGCG
mouse-DMT1/Slc11a2-R	TACTCATATCCAAACGTGAGGG
human-GPX4-F	ATGGTTAACCTGGACAAGTACC
human-GPX4-R	GACGAGCTGAGTGTAGTTTACT
mouse-GPX4-F	TGCTGAGTGTGGTTTACGAATCCTG
mouse-GPX4-R	CGGCTGCAAACTCCTTGATTTCTTG
human-COX2/PTGS2-F	TGTCAAAACCGAGGTGTATGTA
human-COX2/PTGS2-R	AACGTTCCAAAATCCCTTGAAG
mouse-COX2/PTGS2-F	ATTCCAAACCAGCAGACTCATA
mouse-COX2/PTGS2-R	CTTGAGTTTGAAGTGGTAACCG
human-ACSL4-F	ACCAGGGAAATCCTAAGTGAAG
human-ACSL4-R	GGTGTTCTTTGGTTTTAGTCCC
mouse-Acsl4-F	CAATAGAGCAGAGTACCCTGAG
mouse-Acsl4-R	TAGAACCACTGGTGTACATGAC
human-IL-6-F	CACTGGTCTTTTGGAGTTTGAG
human-IL-6-R	GGACTTTTGTACTCATCTGCAC
mouse-IL-6-F	CTCCCAACAGACCTGTCTATAC
mouse-IL-6-R	CCATTGCACAACTCTTTTCTCA
human-IL-10-F	GTTGTTAAAGGAGTCCTTGCTG
human-IL-10-R	TTCACAGGGAAGAAATCGATGA
mouse-IL-10-F	TTCTTTCAAACAAAGGACCAGC
mouse-IL-10-R	GCAACCCAAGTAACCCTTAAAG
human-TNF-α-F	AAGGACACCATGAGCACTGAAAGC
human-TNF-α-R	AGGAAGGAGAAGAGGCTGAGGAAC
mouse-TNF-α-F	ATGTCTCAGCCTCTTCTCATTC
mouse-TNF-α-R	GCTTGTCACTCGAATTTTGAGA
human-IL-1β-F	TGTCGTTGCTTGGTTCTCCTTGTAC
human-IL-1β-R	AGGAGCACTTCATCTGTTTAGG
mouse-IL1-β-F	CACTACAGGCTCCGAGATGAACAAC
mouse-IL1-β-R	TGTCGTTGCTTGGTTCTCCTTGTAC
human-β-actin-F	CCTGGCACCCAGCACAAT
human-β-actin-R	GGGCCGGACTCGTCATAC
mouse-β-actin-F	GTGCTATGTTGCTCTAGACTTCG
mouse-β-actin-R	ATGCCACAGGATTCCATACC

### Luciferase reporter assay

The binding site between the 3’UTR of ACSL4 mRNA and miR-129-5p was predicted by Starbase. Plasmids containing wild-type and mutant 3’UTR of ACSL4 were obtained from Fenghbio. The mimics, mimics-NC, inhibitor, and inhibitor-NC of miR-129-5p and the plasmid carrying ACSL4 3’UTR were co-transfected into HEK293T cells. After 24 h, transfection efficiency and luciferase activity were measured by qRT-PCR and the dual luciferase assay kit (Vazyme).

### TUNEL staining

Colonic cell apoptosis was determined by TUNEL staining. After routine dewax, paraffin sections were incubated with 20 µg/ml proteinase K for 20–30 min before staining according to the instructions. The green fluorescence (FITC) at 520 ± 20 nm and blue fluorescence (DAPI) at 460 nm were observed by fluorescence microscope.

### Immunohistochemical analysis

The tissue in paraffin sections was dewaxed and then blocked with a 3% hydrogen peroxide solution for endogenous peroxidase at room temperature. The antigen was repaired with stimulation buffer by steaming in an electric oven for 30 min, and the non-specific antigen was blocked by incubation with 5% BSA solution. The paraffin sections were then incubated with an anti-ACSL4 antibody (1:200; Affinity) at 4℃ overnight, and the secondary antibody was applied at 37℃ for 30 min. Subsequently, DAB (BOSTER) was used for color development and dehydrated in ethanol. Pictures were acquired using a pathology section scanner.

### Prussian blue staining

Colonic tissues were fixed in 4% paraformaldehyde, dehydrated, and embed. The sections on slides were dewaxed and soaked in Perls Stain (Solarbio) for 25 min. After 1–2 min of washing with distilled water, the samples were immersed in a nuclear solid red dye solution for 5 min and washed. The samples were routinely dehydrated and resin sealed. Pictures were obtained with a pathological section scanner.

### Immunofluorescence analysis

The cells were plated at low density and fixed with 4% paraformaldehyde for 30 min at room temperature. The cells were incubated with Triton X-100 (0.1%) for 30 min to increase the membrane permeability to the antibody and blocked with 5% BSA. They were then incubated with the primary antibody at 4℃ overnight (ACSL4; 1:200, GPX4; 1:200), followed by the fluorescent secondary antibody at 37℃ for 1 h. The slides were sealed with a prolonged live antifade reagent containing DAPI. Images were obtained by a confocal laser microscope (Nikon).

### Hematoxylin and eosin (HE) staining

The mice’s colonic tissues were fixed in 4% paraformaldehyde and embedded in paraffin. Theparaffin-embedded tissue was cut into 4-µm sections, dehydrated in ethanol, and stained with hematoxylin and eosin for light microscopy. Pictures were obtained with a pathological section scanner.

### Statistics

All data were expressed as mean ± SEM. Statistical analysis was performed by using GraphPad Prism software for analysis of variance. Student’s t-test was performed for comparisons between any two groups. One-way analysis of variance (ANOVA) followed by Tukey’s multiple comparison tests was performed for multiple groups. P ≤ 0.05 was considered statistically significant.

## Results

### Characterization of hucMSC-Ex

**Fig. 1:**
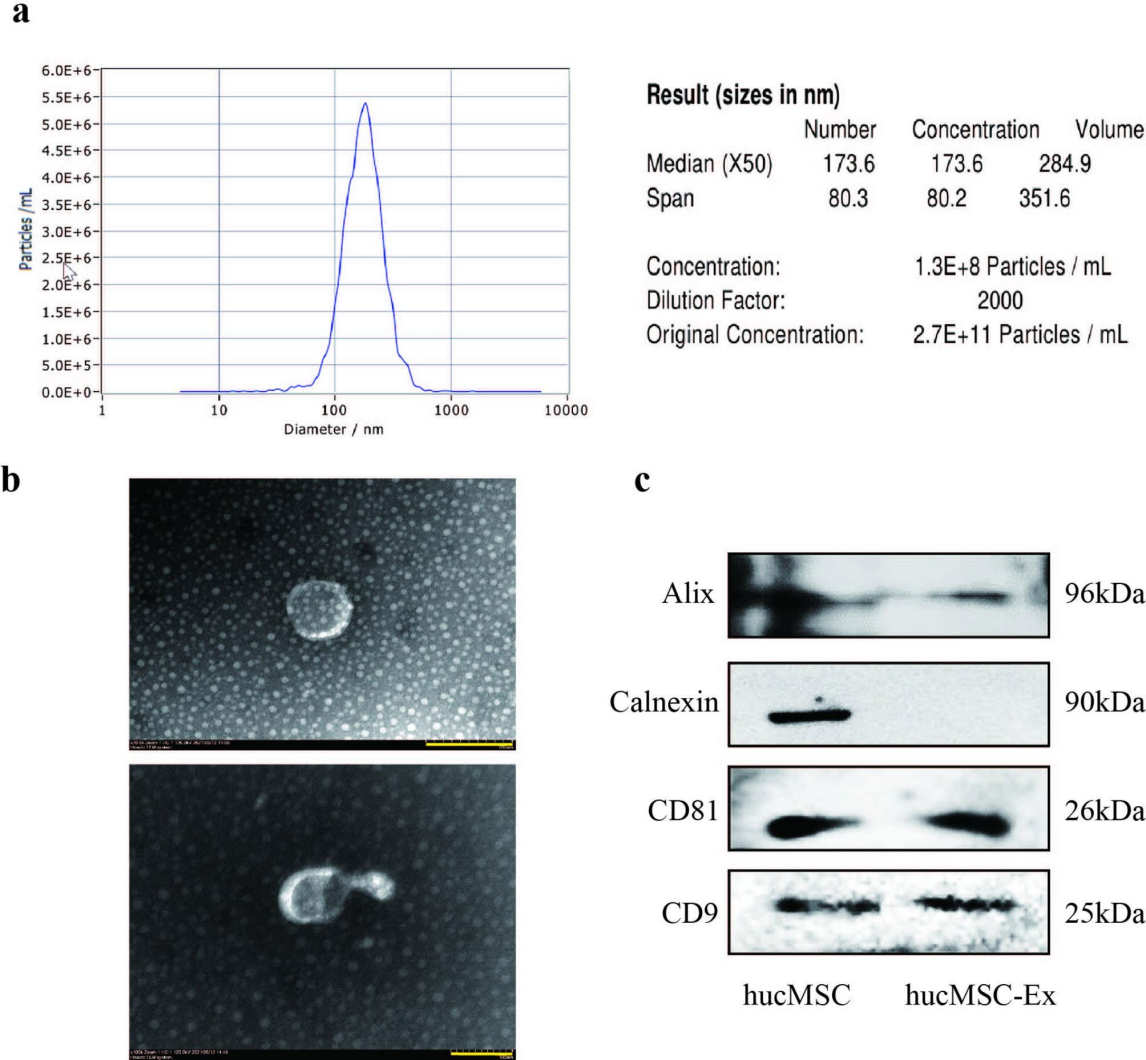
Identification of hucMSC-Ex. **A**, the detection of the size and number of hucMSC-Ex by using a nanoparticle tracking analyzer (NanoSight); **B**, Identification of hucMSC-Ex by transmission electron microscopy (TEM). The scale of the top picture is 200 nm and the scale of the bottom picture is 100 nm; **C**, Identification of hucMSC-Ex surface markers by western blot

We successfully isolated hucMSC from the human umbilical cord for culture and collected hucMSC-Ex by ultracentrifugation. The particle concentration and size of hucMSC-Ex were analyzed by a nanoparticle tracking analyzer (NanoSight). The results showed hucMSC-Ex concentration of 3.7 × 10^11^particles/mL and particle size between 30 and 300 nm (Fig. 1A). The transmission electron microscope indicated that hucMSC-Ex had a low-density electron component in the cell, with a complete vesicular structure in the cell membrane (Fig. 1B). Recent research discovers that tetraspanin and integrin proteins CD9 and CD81 are associated with exosome adhesion targeting [34], and ALG2-interacting protein X (ALIX) is involved in the formation of intraluminal vesicles (ILVs) [35]. They are considered to be the identification markers of exosomes. Western blot results showed that both hucMSC and hucMSC-Ex expressed CD9, Alix, and CD81, while only hucMSC expressed Calnexin (Fig. 1C).

### Validation of hucMSC-Ex repair ability in DSS-induced IBD mice

**Fig. 2:**
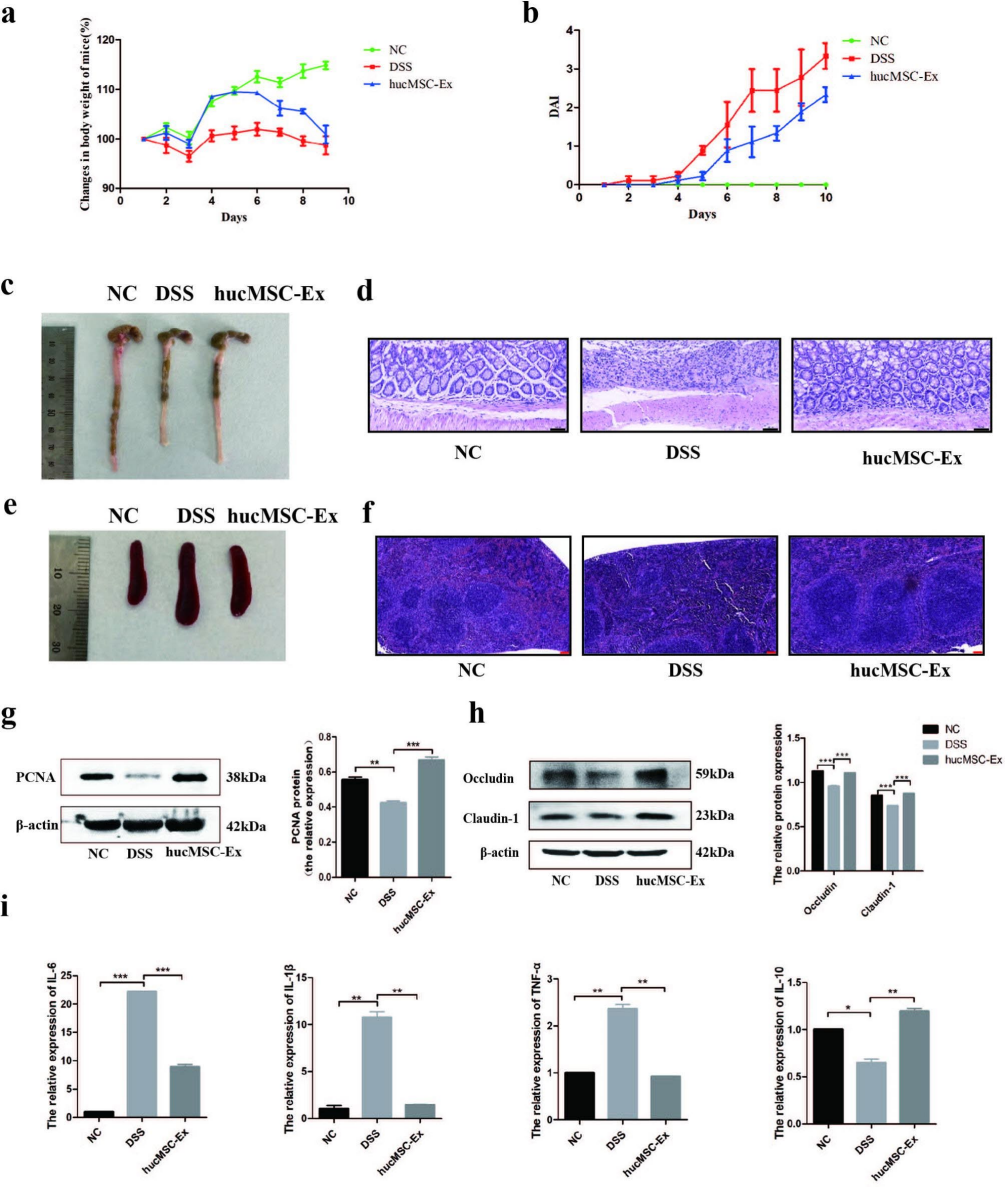
hucMSC-Ex repairs IBD in mice. **A**, Changes in body weight of mice; **B**, Assessment of DAI score of mice; **C**, Gross view of the colon of mice; **D**, **H**&**E** staining of the colon (scale bar = 50 μm); **E**, Gross view of the spleen of mice; **F**, **H**&**E** staining of spleen tissue of mice (scale bar = 100 μm); **G**, Western blot analysis of the expression level of PCNA; **H**, Western blot analysis of the expression level of intestinal tight junction related molecules in colon tissue; **I**, QRT-PCR analysis of inflammatory factors mRNA expression in mice colon tissue. (Data are presented as mean ± SD by student t-test. (^*^P < 0.05; ^**^P < 0.01; ^***^P < 0.001)

To verify the repair effect of hucMSC-Ex on IBD mice, 3% DSS was used to induce IBD, and hucMSC-Ex was injected via the tail vein. The results showed that the weight of the mice in the NC group increased over time, while the weight of the DSS group did not. The hucMSC-Ex treated group increased in weight with time but began steadily decreasing on the sixth day (Fig. 2a). Mice in the DSS group had severe diarrhea accompanied by bloody stools. After the hucMSC-Ex injection, diarrhea and bloody stools were relieved and the DAI score decreased, indicating signs of IBD mitigation in mice (Fig. 2b). After the mice were sacrificed on the 10th day, colons and spleens were taken for examination. Results demonstrated that the spleen and colon of DSS mice were enlarged (indicating the occurrence of an immune overreaction) and shortened, respectively, while hucMSC-Ex treatment significantly restored spleen size and colon length. (Figure 2c and e). To examine the effect of the treatment on intestinal cell proliferation and tight junction integrity, the proliferation marker PCNA (proliferating cell nuclear antigen) and tight junction molecules Occludin and Claudin-1 were measured. Compared to the DSS group, the hucMSC-Ex showed increased protein levels of PCNA, Occludin, and Claudin-1 (Fig. 2g h). Moreover, H&E staining results showed that the intestinal mucosal structure was distorted and the spleen tissue integrity of mice in the DSS group was damaged by inflammatory cell infiltration, while the colonic structure and spleen tissue integrity were markedly restored in the hucMSC-Ex group. (Figure 2d and f), indicating the effective repair ability of hucMSC-Ex on DSS-induced IBD in mice. In addition, qRT-PCR was used to analyze inflammatory factors in the colon tissue. The results showed that the mRNA expression levels of pro-inflammatory factors IL-6, TNF-α, and IL-1β in the DSS mice were significantly increased, while the anti-inflammatory factor IL-10 was decreased. The hucMSC-Ex reversed this trend by downregulating IL-6, TNF-α, and IL-1β, and upregulating IL-10, which shows that hucMSC-Ex has an anti-inflammatory repair effect on IBD in mice from the molecular level (Fig. 2i).

### HucMSC-Ex repaired IBD by inhibiting ferroptosis in mice colon tissues

**Fig. 3:**
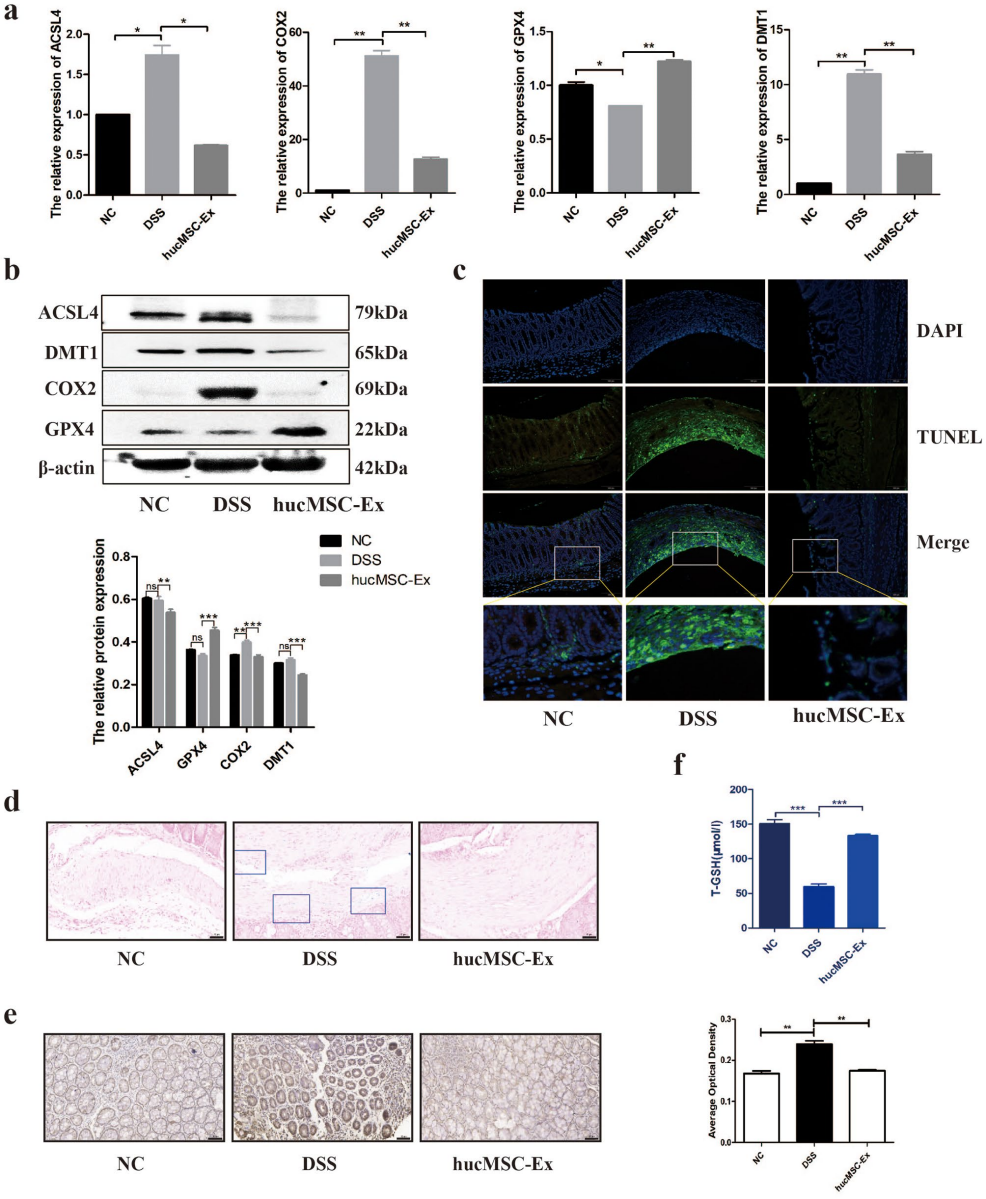
hucMSC-Ex repairs IBD by inhibiting ferroptosis in the colon of mice. **A**, Analysis of mRNA expression level of ferroptosis-related genes in mice colon by qRT-PCR; **B**, Western blot analysis of the expression level of ferroptosis-related molecules in colon tissue of mice; **C**, TUNEL staining of colon tissues of mice (scale bar = 100 μm); **D**, Prussian blue staining of colon tissues of mice (scale bar = 50 μm); **E**, IHC analysis of colon tissue level of ACSL4 and the average optical density (scale bar = 50 μm); **F**, Total GSH content in the colon of mice. Data are presented as mean ± SD by student t-test. *P < 0.05; **P < 0.01; ***P < 0.001.

Next, we analyzed the ferroptosis-related indicators of mice colon tissues to explore whether hucMSC-Ex can regulate ferroptosis. The results of qRT-PCR showed that the mRNA expression levels of divalent metal transporter 1 (DMT1), cyclooxygenase 2 (COX2), and ACSL4 significantly increased but GPX4 decreased in the DSS group, while hucMSC-Ex treatment downregulated DMT1, COX2, and ACSL4 and upregulated GPX4 (Fig. 3a). Since these genes are related to ferroptosis, the result suggests that hucMSC-Ex plays a corresponding protective role in the regulation of ferroptosis, at least in inhibiting ferroptosis in some related pathways. Western blot results were consistent with the mRNA expression level, which confirms the role of hucMSC-Ex in ferroptosis (Fig. 3b). TUNEL staining showed apoptotic cells with green fluorescence, where apoptotic cells in the colon tissue of IBD mice increased significantly but decreased in the hucMSC-Ex group (Fig. 3c). Prussian blue staining indicated an accumulated deposition of small iron particles (in blue frame) in the colon tissue of IBD mice, indicating a possible iron transport or metabolism disorder in the intestine of IBD mice. However, after hucMSC-Ex treatment, iron particle deposition was significantly reduced (Fig. 3d). Immunohistochemical was used to further confirm the increased ACSL4 expression in the colon epithelium of IBD mice compared to the reduced level in mice treated with hucMSC-Ex (Fig. 3e). In addition, the colonic mucosal tissues of the mice were extracted and weighed, and tissue homogenate was prepared according to the ratio of the weight; dilution = 1:4. The total glutathione content (T-GSH) of the colonic tissues of the mice was determined. Results showed that while T-GSH was depleted in the colonic tissues of IBD mice, it increased significantly after hucMSC-Ex treatment. (Fig. 3f). The results presented above suggest that hucMSC-Ex may repair IBD by inhibiting lipid peroxidation and, consequently, the iron-related destruction of intestinal epithelial cells.

### HucMSC-Ex inhibits lipid peroxidation and alleviates ferroptosis in intestinal epithelial cells in vitro

**Fig. 4:**
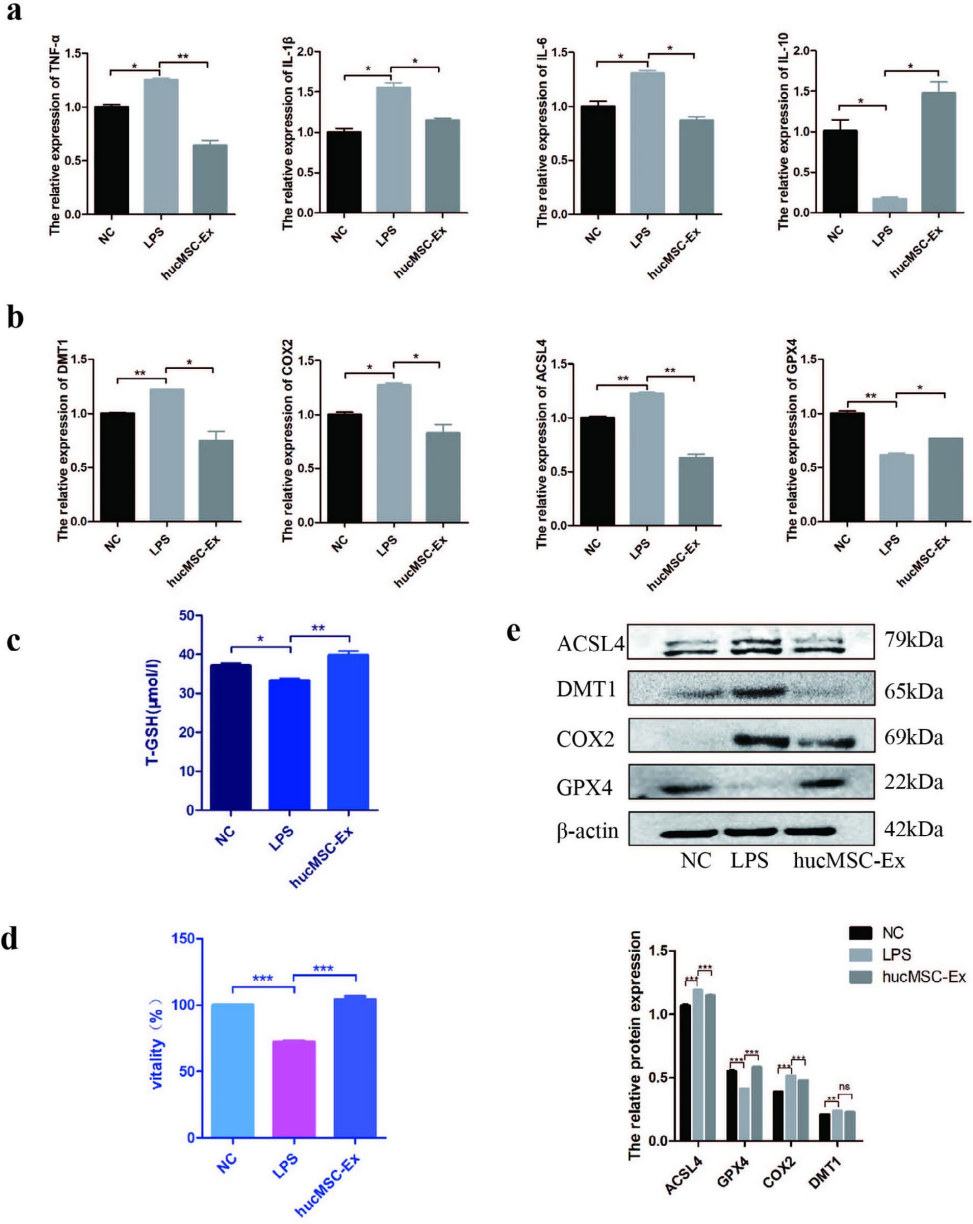
hucMSC-Ex inhibits lipid peroxidation in vitro to reduce ferroptosis and repair IBD. **A**, QRT-PCR analysis of inflammatory factors mRNA expression in HCoEpiC; **B**, Analysis of the mRNA expression level of ferroptosis-related genes in HCoEpiC by qRT-PCR; **C**, Total GSH content in HCoEpiC; **D**, Cell viability assay; **E**, Western blot analysis of the expression level of ferroptosis-related molecules in HCoEpiC. Data are presented as mean ± SD by student t-test.*P < 0.05; **P < 0.01; ***P < 0.001

After determining that ACSL4 is highly expressed in the intestinal epithelial cells of IBD mice, promoting LPO and iron transport/metabolism disorder, we selected human normal colon epithelial cell line HCoEpiC to verify the repair effect of hucMSC-Ex on IBD in vitro. An in vitro inflammatory model was established using LPS induction and treated with hucMSC-Ex. After LPS treatment, the expressions of pro-inflammatory factors TNF-α, IL-6, and IL-1β were increased, while the expression of anti-inflammatory factor IL-10 was decreased, and the trend was reversed after hucMSC-Ex treatment (Fig. 4a). The application of LPS triggered the up-regulation of the ferroptosis-related molecules ACSL4, DMT1, and COX2, and down-regulation of GPX4. Similar to the observation in the in vivo study, hucMSC-Ex treatment inhibited the levels of ACSL4, DMT1, and COX2, but significantly restored the depletion of GPX4 (Fig. 4b). The depletion of T-GSH is related to GPX4. By measuring T-GSH in cells, we found that the amount of T-GSH positively correlates with the trend of GPX4 expression (Fig. 4c). Further analysis showed that LPS decreased the survival of intestinal epithelial cells, while treatment with hucMSC-Ex significantly increased the survival of the cells (Fig. 4d). In an in vitro validation of LPO in intestinal epithelial cells, we found that the expression of ACSL4 protein and mRNA was upregulated in the LPS-induced inflammatory environment, confirming the activation of LPO-related processes in IBD (Fig. 4b, e). Our results validate the anti-inflammatory effect of hucMSC-Ex in vitro and its ability to inhibit LPO, thereby reducing ferroptosis cell death and repairing IBD.

### HucMSC-Ex derived miR-129-5p targets ACSL4 to inhibit lipid peroxidation and repair IBD

**Fig. 5:**
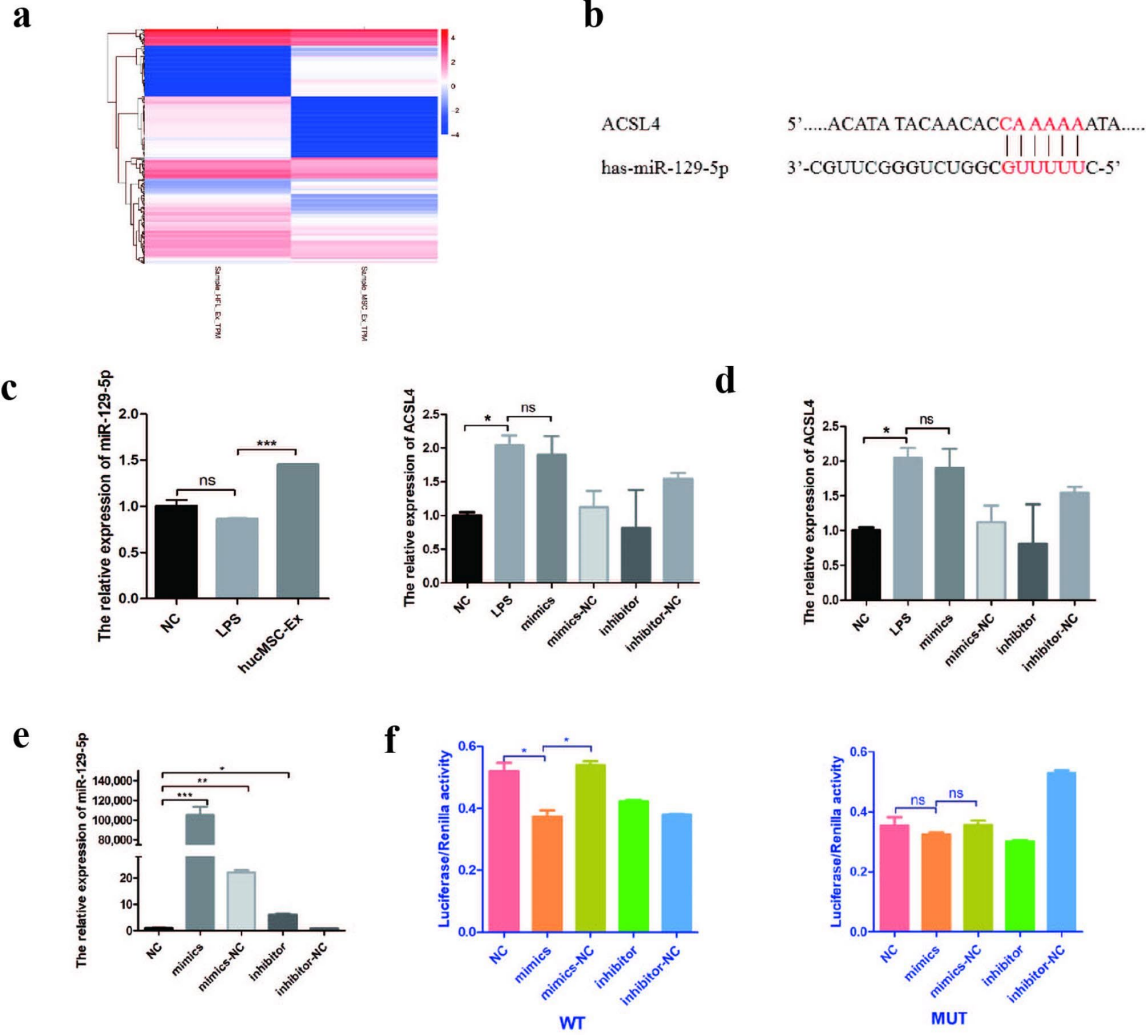
miR-129-5p is highly expressed in hucMSC-Ex. **A**, small RNA sequencing results of hucMSC-Ex; **B**, The binding site of miR-129-5p to the 3’-UTR of ACSL4 mRNA sequence; **C**, The expression of miR-129-5p in HCoEpiC co-incubated with hucMSC-Ex; **D**, Expression level of ACSL4 mRNA after transfection of miR-129-5p mimics and inhibitors; **E**, Detection of transfection efficiency of miR-129-5p mimics and inhibitors; **F**, Dual-luciferase reporter gene of the targeting relationship between miR-129-5p and ACSL4. Data are presented as mean ± SD by student t-test. *P < 0.05; **P < 0.01; ***P < 0.001

Studies have shown that hucMSC-Ex acts as an anti-inflammatory and repair agent by serving as a medium for information exchange between cells. Our research group conducted small RNA sequencing on hucMSC-Ex and HFL1-Ex, and found that miR-129-5p was highly expressed in hucMSC-Ex (Fig. 5a source: Key Laboratory of Medical Science and Laboratory Medicine of Jiangsu Province, School of Medicine, Jiangsu University). Based on the observed effect of hucMSC-Ex on ferroptosis-related molecules (Fig. 4), we selected the key ferroptosis enzyme ACSL4, screened the miRNAs in the database for which it had an effect, and compared them to our sequencing data. As predicted by the Starbase database (https://starbase.sysu.edu.cn/index.php), miR-129-5p has the ability to target ACSL4 and we hypothesize that it can influence the expression of the ACSL4 protein (Fig. 5b). After repeating qRT-PCR verification, the expression of miR-129-5p was up-regulated in the hucMSC-Ex treatment group, indicating that hucMSC-Ex carries and transports miR-129-5p into intestinal epithelial cells to participate in its role (Fig. 5c). Moreover, the miR-129-5p mimics alone did not significantly affect the expression of the ACSL4 mRNA, suggesting that miR-129-5p may play a role in translation progression. (Fig. 5d). Based on these observations, miR-129-5p mimics and inhibitors were constructed and transfected into HCoEpiC to verify the transfection efficiency. The results showed that miR-129-5p mimics were successfully transferred into the intestinal epithelial cells (Fig. 5e). To further verify the binding of miR-129-5p and ACSL4, we constructed plasmids containing ACSL4 3’UTR and mutant 3’UTR, and co-transfected 293T with miR-129-5p. The results confirmed that the ratio of luciferase activities of the WT in the miR-129-5p mimics group was significantly reduced. In contrast, the activity ratio of the inhibitor group increased (Fig. 5f). Our results indicated that miR-129-5p had binding sites on the 3’UTR of ACSL4.

### Verification of miR-129-5p effect on ferroptosis in vitro

**Fig. 6:**
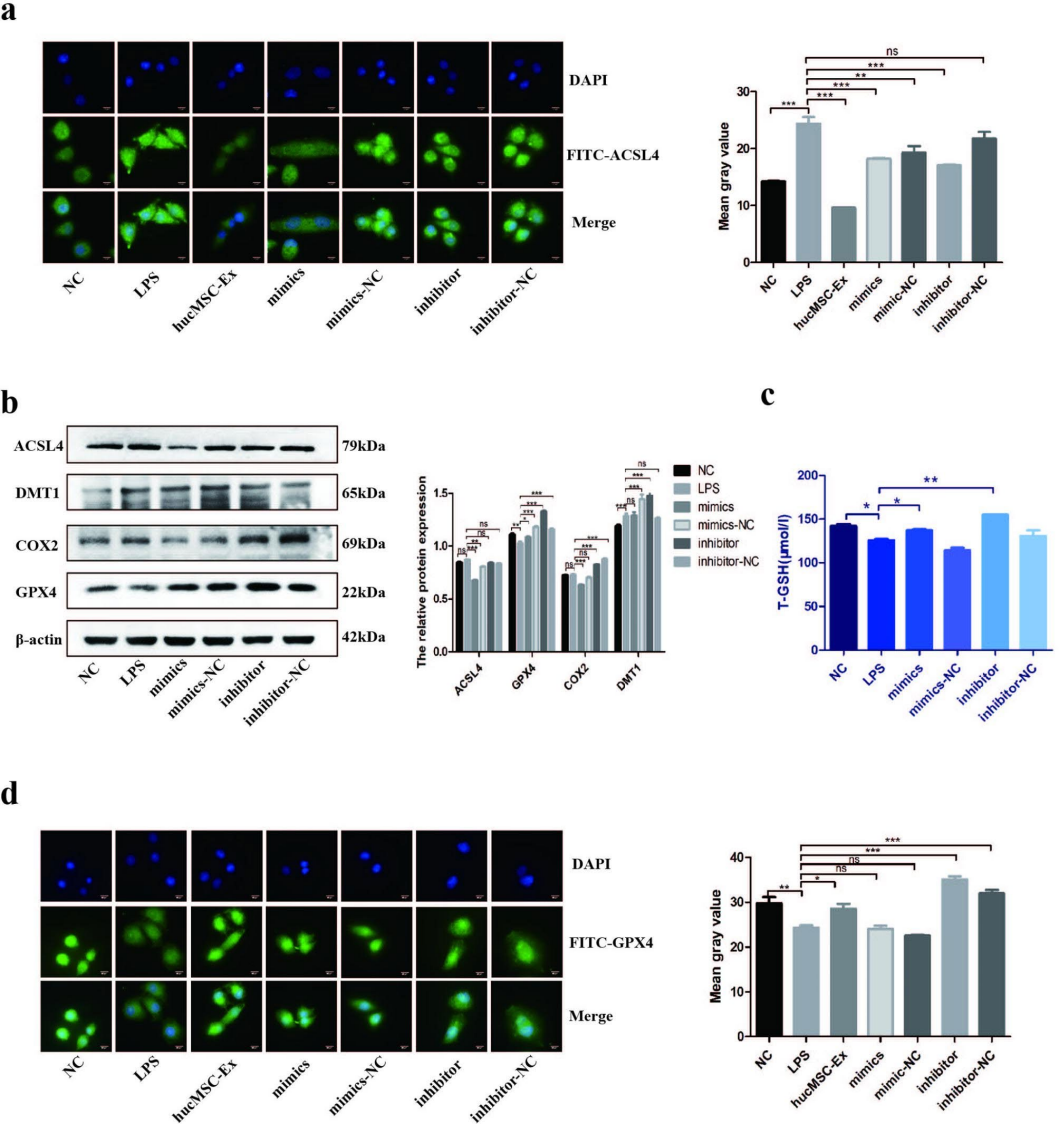
Verification of the effect of miR-129-5p on ferroptosis in vitro. **A**: Immunofluorescence detection of ACSL4 expression in HCoEpiC and the mean grayscale value; **B**: Detection of ferroptosis-related molecules by western blot; **C**: T-GSH content of HCoEpiC; **D**, The expression of GPX4 in HCoEpiC by immunofluorescence and the mean grayscale value; Data are presented as mean ± SD by student t-test. *P < 0.05; **P < 0.01; ***P < 0.001

After identifying the relationship between miR-129-5p and ACSL4, we verified the relationship between miR-129-5p and ferroptosis in HCoEpiC. We transfected HCoEpiC with miR-129-5p mimics, inhibitors, or hucMSC-Ex, and compared their effects on LPS-induced ferroptosis. The results of immunofluorescence showed that the fluorescence intensity of ACSL4 protein in hucMSC-Ex and miR-129-5p mimics groups was inhibited, while the miR-129-5p mimics-NC, inhibitor, and inhibitor-NC groups were the same as that in LPS group, which indicated that hucMSC-Ex and miR-129-5p had the same inhibitory effect on the expression of ACSL4 protein (Fig. 6a). Next, we explored whether miR-129-5p could affect the expression of other molecules related to ferroptosis. Western blot results showed that the transfer of miR-129-5p mimics could inhibit the expression of ACSL4 protein, reverse the depletion of GPX4, and reduce the expression of COX2, but had no effect on DMT1 (Fig. 6b). The detection of T-GSH indicates that miR-129-5p increases T-GSH expression and, therefore, increases the antioxidant capacity of the cell (Fig. 6c). Immunofluorescence of GPX4 protein further verified that hucMSC-Ex and miR-129-5p have similar effects on ferroptosis (Fig. 6d).

### HucMSC-Ex serves as a nanocarrier of mir-129-5p to inhibit DSS-induced IBD in mice

**Fig. 7:**
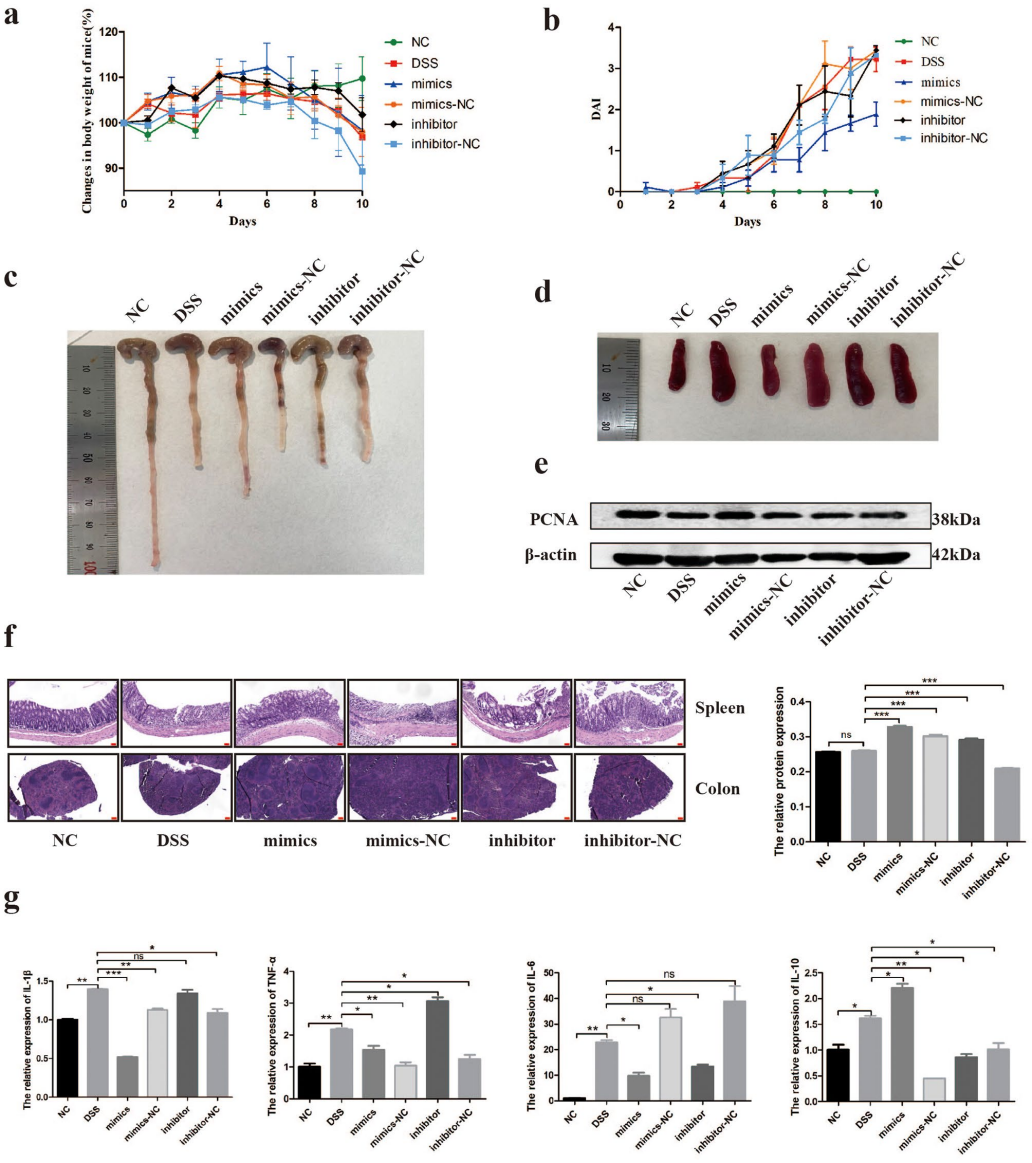
Repair of DSS-induced IBD by hucMSC-Ex transfected with miR-129-5p mimics and inhibitors. **A**, Changes in body weight of mice; **B**, Assessment of DAI score of mice; **C**, Gross view of mice colon; **D**, Gross view of mice spleen; **E**, Western blot analysis of the expression level of PCNA in mice; **F**, H&E staining of mice colon (scale bar =50 μm) and spleen (scale bar = 200 μm); **G**, Expression levels of inflammatory factors in mice intestinal tissue. Data are presented as mean ± SD. *P < 0.05; **P < 0.01; ***P < 0.001

After the in vitro inhibitory effect of miR-129-5p on ferroptosis was verified, we wondered whether hucMSC-Ex played a role in alleviating IBD through miR-129-5p. We used hucMSC-Ex as a nanocarrier, transfected miR-129-5p into hucMSC-Ex using an exosome transfection kit, and injected hucMSC-Ex carrying miR-129-5p mimics and inhibitors into DSS induced IBD mice via tail vein. The results showed that the body weight of mice in the NC group increased steadily while that of the DSS group decreased significantly at the later stage. The body weight of the mimics group increased steadily up to day 6 and began to decrease at the last stage of the model. Weight gain in the mimics-NC and inhibitor groups was similar to that in the mimics group, while weight loss was significant in the inhibitor-NC group (Fig. 7a). DAI scores showed that the mice in the mimics group were in good condition, while those in the other groups were in poor condition (Fig. 7b). Colon length significantly increased and spleen size was reduced in the mimics group, but shortened and enlarged in the other groups, respectively (Fig. 7c and d). Western blot results showed that the expression of PCNA protein in the colon of mice in the mimics group was up-regulated while the expression in the other groups was unchanged compared to the DSS group (Fig. 7e). H&E staining showed signs of colon repair, with clear glandular structure and reduced inflammatory cell infiltration in the mimics group. In the DSS and other transfection groups, the intestinal structure was disordered with increased inflammatory cell infiltration (Fig. 7f). Similarly, H&E staining of the spleen showed destroyed splenic nodules in the DSS and other groups, but intact in the mimics group (Fig. 7F). Moreover, the analysis of the expression of inflammatory factors in the colon tissue by qRT-PCR showed that IL-6, TNF-α, and IL-1β in the mimics group were downregulated and IL-10 was upregulated (Fig. 7g). Since the vector is hucMSC-Ex, inflammatory factors in the other transfection groups may decline, but the downtrend may not be as strong as that in mice in the mimics group.

**Fig. 8:**
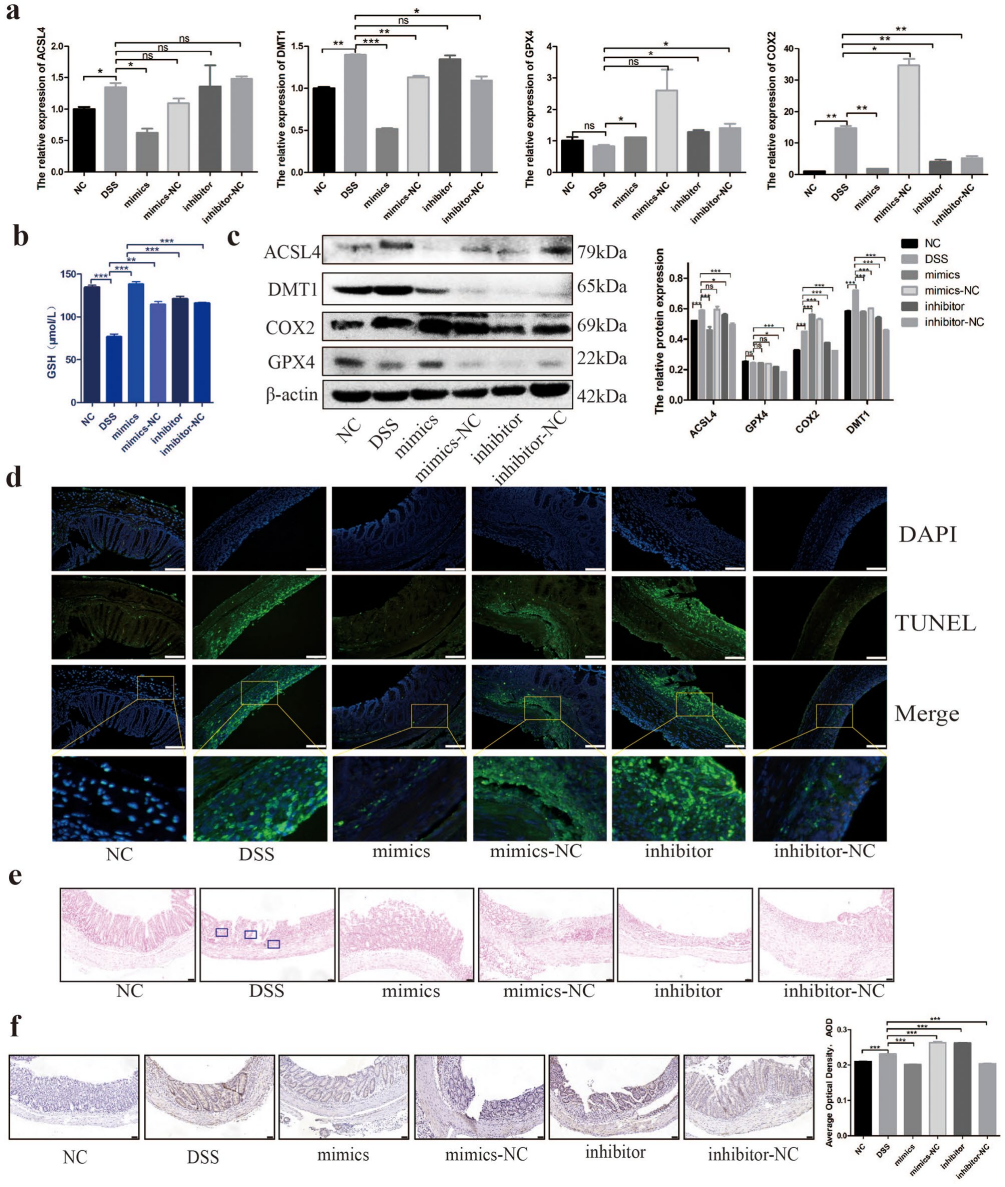
hucMSC-Ex transfected with miR-129-5p targets ACSL4 to inhibit ferroptosis in DSS-induced IBD mice intestinal epithelial cells. **A**, The expression level of ferroptosis-related molecular in mice intestinal tissue; **B**, T-GSH concentration in mice colon tissue; **C**, The expression level of ferroptosis-related molecules in mice colon by western blot; **D**, TUNEL staining (scale bar = 100 μm); **E**, Prussian blue staining of mice intestinal tissue (scale bar = 50 μm); **F**, The expression of ACSL4 as detected by immunohistochemistry and the average optical density (scale bar = 50 μm). Data are presented as mean ± SD. *P < 0.05; **P < 0.01; ***P < 0.001

After demonstrating that miR-129-5p could alleviate DSS-induced IBD in mice using hucMSC-Ex as the vector, we further explored the effects of hucMSC-Ex transfected with miR-129-5p mimics and inhibitors on LPO and ferroptosis. We first analyzed the molecules related to ferroptosis in the colon tissues of each group of mice. The results were similar to in vitro experiments, as the miR-129-5p mimics group could significantly reduce the mRNA expression levels of DMT1, COX2, and ACSL4 and reverse the depletion of GPX4, while the other transfection groups increased the ferroptosis-related molecules compared to the mimics group (Fig. 8a). In addition, the western blot showed that the expression of ACSL4 protein, the target molecule of miR-129-5p, was significantly decreased in the mimics group compared with the other groups. GPX4 depletion was also significantly reversed only in the mimics group, and DMT1 was downregulated in all transfection groups (Fig. 8c). T-GSH expression by qRT-PCR was decreased in the DSS group but recovered in all transfection groups, however, apart from the mimics group, this was inconsistent with the western blot results, which might be due to other regulatory factors of this protein expression (Fig. 8b and c). TUNEL staining of intestinal tissue sections showed that apoptosis of intestinal cells increased in the DSS group but only decreased significantly in the miR-129-5p mimics group (Fig. 8d). Moreover, iron staining indicated scattered iron particle deposits in the colons of mice in the DSS group but was occasionally observed in other transfection groups, indicating that all hucMSC-Ex transfection groups had the ability to reduce iron accumulation (Fig. 8e). To further verify the expression of ACSL4, immunohistochemical staining was performed and the results showed that hucMSC-Ex carrying miR-129-5p could significantly reduce the expression of ACSL4, while the other transfection groups could only slightly reduce the expression of ACSL4 (Fig. 8f).

## Discussion

In this study, we explored the repair effect of hucMSC-Ex on IBD in vivo and in vitro and demonstrated that hucMSC-Ex inhibits LPO by reducing the markers of ferroptosis to repair IBD. In our previous studies, we reported that hucMSC-Ex, an emerging cell-free therapy, shows a strong IBD repair effect [36–38]. Regardless, how hucMSC-Ex effectively performs its inflammation-suppressing and tissue-repairing functions are constantly being explored. Iron metabolism disorders have been discussed in the occurrence and development of IBD, and inhibition of ferroptosis has also been noted by researchers as a therapeutic target for IBD [33, 39]. Researchers found that endoplasmic reticulum stress signals can induce ferroptosis in intestinal epithelial cells, and phosphorylation of NF-κB signals can alleviate ferroptosis [40]. Other studies have also shown that in DSS-induced mouse models, ACSL4 and COX2 are significantly upregulated while GPX4 is decreased. Treatment with ferroptosis inhibitors ferrostatin-1, liproxstatin-1, or deferprone, reverse this trend and alleviates DSS-induced enteritis in mice, and is related to blockade of the Nrf2/HO-1 signaling pathway [41]. In this study, hucMSC-Ex, as a promising therapy for IBD, was analyzed for its effect on intestinal ferroptosis. The mRNA and protein expression levels of ACSL4, DMT1, COX2, and GPX4 in mice colon and an intestinal epithelial cell line were analyzed to determine whether there was an iron transport disorder and abnormal LPO in the inflammatory environment of IBD models. Results indicated the ability of hucMSC-Ex to regulate key drivers of ferroptosis, indicating that hucMSC-Ex plays a role in inflammation repair by inhibiting intestinal ferroptosis. This result is consistent with the results obtained by other researchers, indicating that IBD co-exists with ferroptosis and that the inhibition of ferroptosis alleviates IBD [32, 42].

LPO is a key marker of ferroptosis, where PUFAs serve as the substrate and ACSL4 mediates the synthesis of phospholipids from CoA and PUFAs [23, 43]. Several studies report that the intake of PUFAs in a high-fat diet is positively correlated with the occurrence of IBD, however, few studies have elaborated on the direct relationship between ACSL4 and IBD [44, 45]. Our data, both in vivo and in vitro, confirm that intestinal inflammation is associated with elevated ACSL4. In addition, intestinal inflammation is accompanied by the inactivation of the antioxidant system represented by GSH and GPX4. GPX4 mainly reduces lipid hydroperoxides in cell membranes to non-toxic lipid alcohols. Generally, GPX4 is deactivated after GSH depletion, constituting the breakdown of the antioxidant system, which is one of the mechanisms that promote ferroptosis [23, 46]. Our results mainly highlight the inhibition of the LPO process. The reduced expression of ACSL4, a key enzyme in lipid peroxidation, and the increased expression of GPX4 and GSH, key molecules that inhibit the lipid peroxidation process, after hucMSC-Ex treatment, implicate downregulated LPO. Moreover, the upregulation of COX2 is associated with oxidative stress, a promoter of lipid peroxidation, and COX2 colocalizes with 4-hydroxy-2-nonenal (HNE), a major lipid peroxidation-derived aldehyde [47]. HNE also induces COX2 expression and is likely linked with oxidative stress and chronic inflammation through the activation of cyclooxygenase [48]. Thus, the ability of hucMSC-Ex to reduce COX2 and ACSL4 enhances tissue repair by reducing ferroptosis.

Iron overload has long been reported in alcoholic liver disease, cardiomyopathy, and spinal cord injury [49–51]. It has also been suggested that excess iron supplementation may increase inflammation in the gut, as excessive iron aggravates the inflammatory reaction or cause epigenetic changes in intestinal epithelial cells [52, 53]. Our results indicate that DSS-induced increased intestinal iron accumulation and high expression of the iron transporter DMT1 in mice intestinal mucosa and HCoEpiC. In addition, we have demonstrated that there are iron transport obstacles, exhaustion of antioxidant systems, and activation of lipid peroxidase related to ferroptosis in IBD mice, and hucMSC-Ex has a strong ability to reverse the overall progress of these changes. HucMSC-Ex inhibits ferroptosis and alleviates intestinal inflammation, but the specific mechanism needs further exploration.

We also examined how hucMSC-Ex functions as an inhibitor of ferroptosis in IBD. Exosomes are nanoscale vesicles containing a variety of bioactive substances, which have been shown to activate certain adenosine, mediate the activity of related pathways, and inhibit the expression of certain inflammatory factors [7, 54]. Researchers have found that small RNAs contained in MSC-Ex are one of the important mediators for the paracrine role of exosomes [55, 56]. MSC-Ex can regulate macrophage polarization via miR-182 and inactivate TLR4/NF-κB/PI3K/Akt signaling cascades to alleviate myocardial ischemia/reperfusion (I/R) injury [15]. Cheng et al. demonstrated that the beneficial effects offered by MSC-Ex transplantation after myocardial infarction are because of excreted exosomes containing mainly miR-210 [57]. Song et al. found that hucMSC-Ex inhibits ferroptosis and reduces myocardial injury by delivering miR-23a-3p [58]. This proves that the miRNA family contributes to the biological role of hucMSC-Ex. Our results demonstrate for the first time the relationship between miR-129-5p and ferroptosis inhibition. By small RNA sequencing, we identified miR-129-5p contained in hucMSC-Ex and predicted its binding to ACSL4. To verify the effect of miR-129-5p on the LPO of intestinal epithelial cells, we constructed mimics and inhibitors. First, we verified the effect of miR-129-5p in vitro and determined that it can reduce the protein expression of ACSL4. Next, we verified the effect of miR-129-5p in mice, by transferring miR-129-5p into hucMSC-Ex and administrating it to IBD mice. Our results indicate that hucMSC-Ex transfected with miR-129-5p could also inhibit ferroptosis by decreasing the expression of ACSL4, COX2, and DMT1 while increasing GPX4 and GSH in vivo. Regardless of these promising observations, certain experimental challenges still need to be resolved. Firstly, since hucMSC-Ex is a complex bio-derived vesicle containing many bioactive substances, including thousands of small RNAs and enzymes with strong biological activities, we cannot guarantee that the results obtained are only due to the action of miR-129-5p; the effect may be the combined action of other bioactive molecules in hucMSC-Ex and miR-129-5p. Secondly, since the transfer of mimics makes the concentration of miR-129-5p in hucMSC-Ex rise sharply, there is a need for further studies on the appropriate proportion to obtain the best effect. Finally, this study serves as the first data on hucMSC-Ex mitigation of IBD via inhibiting ferroptosis, thus providing a basis for more mechanistic exploration in future studies.

## Conclusion

In conclusion, our results demonstrate that hucMSC-Ex relieves IBD by targeting ACSL4 with miR-129-5p to reduce LPO levels and ferroptosis, reducing intestinal inflammation and repairing the damage.

## Data Availability

All data in this study are available from the corresponding author upon reasonable request.
